# Findings Related to Cerebrospinal Fluid and Central Nervous System Disorders in Small Ruminants—A Retrospective Study on Sheep and Goats

**DOI:** 10.3390/ani14010046

**Published:** 2023-12-21

**Authors:** Leandra C. Schöb, Christian Gerspach, Martina Stirn, Regina Hofmann-Lehmann, Barbara Riond

**Affiliations:** 1 Clinical Laboratory, Department for Clinical Services and Diagnostics, Vetsuisse Faculty, University of Zurich, 8057 Zurich, Switzerland; leandrachristina.schoeb@uzh.ch (L.C.S.); martina.stirn@roche.com (M.S.); rhofmann@vetclinics.uzh.ch (R.H.-L.); 2 Clinic for Ruminants, Department of Farm Animals, Vetsuisse Faculty, University of Zurich, 8057 Zurich, Switzerland; cgerspach@vetclinics.uzh.ch

**Keywords:** CSF analysis, small ruminants, sheep, goat, CNS diseases, cytology, pleocytosis, listeriosis

## Abstract

**Simple Summary:**

Globally, small ruminants are important animals since they provide food, income, and livelihoods for millions of people, contributing to biodiversity conservation and offering a range of products with cultural, economic, and environmental significance. Central nervous system (CNS) diseases in sheep and goats encompass a range of conditions that affect the brain and spinal cord. The diseases afflicting these animals can have significant economic and health implications. The diagnosis and management of CNS diseases affecting small ruminants often require the involvement of veterinary care. The timely identification and treatment of these conditions are essential to minimizing the impact on the affected animals and preventing the spread of contagious diseases to herds or flocks. Analysis of cerebrospinal fluid can be an important tool in the diagnosis of CNS diseases in small ruminants, and knowledge about the results of CSF analysis in relation to CNS diseases is of importance and can contribute to improving the health statuses of these animals and humans. The present study investigated the cytological pattern of cerebrospinal fluid in sheep and goats with CNS diseases. Infectious diseases of bacterial origin were determined to be the most common underlying causes for CSF alterations in sheep and goats, followed by parasitic disorders. Alterations in the cellular components of cerebrospinal fluid in small ruminants with CNS disease were mainly due to an increase in monocytic and lymphocytic cells with variable quantitative expression, whereas neutrophilic pleocytosis and cytoalbuminologic dissociation were rare findings.

**Abstract:**

Background: Small ruminants often suffer from central nervous system (CNS) disorders, and cerebrospinal fluid (CSF) analysis can be used as a diagnostic tool in this regard. In small animals and cattle, specific CSF patterns have been defined for specific disease categories. No data exist regarding CSF results obtained from small ruminants and their association with certain CNS diseases. Objectives: The objective of this study was to retrospectively investigate CSF findings obtained from sheep and goats and to identify possible CSF patterns associated with disease categories. Methods: CSF samples and medical records from 44 sheep and 27 goats were included in this study. All animals were presented to the Veterinary Teaching Hospital Zurich of the Veterinary Teaching Hospital Zurich of the Vetsuisse Faculty of the University of Zurich between 2003 and 2016 and had either a confirmed CNS diagnosis or showed CSF changes without a specific CNS diagnosis. Results: Mixed mononuclear pleocytosis was the most common CSF pattern in sheep (25%), followed by monocytic pleocytosis (21%). Lymphocytic pleocytosis was most frequently found in goats (37%). In 75% of sheep and 56% of goats, infectious CNS diseases were diagnosed, with listeriosis being the most common infectious disease in both species, followed by parasitic disorders (nematodiasis and coenurosis). Conclusions: The cytologic CSF patterns in small ruminants are mainly based on the increased presence of monocytic and lymphocytic cells with variable quantitative expression, whereas neutrophilic pleocytosis and cytoalbuminologic dissociation were rare findings. Infectious diseases of bacterial origin were the most common underlying causes for CSF alterations in sheep and goats, followed by parasitic disorders. The pleocytosis type is not helpful for differentiating disease types.

## 1. Introduction

Approximately 355,000 sheep and more than 80,000 goats live in Switzerland [1]; therefore, both ovine and caprine patients constitute a major part of the patient population of farm animal clinics. 

In addition to other diseases, small ruminants often suffer from central nervous system (CNS) disorders, which can be classified as infectious (e.g., bacterial, viral, or parasitic), non-infectious (e.g., metabolic, degenerative, traumatic, or neoplastic), or inflammatory diseases. Many diseases affect a specific location in the CNS, meaning that certain parts of the brain or the spinal cord can be affected to a greater extent. Depending on the pathogen and the type of infection or lesion in the CNS, changes in the cellular composition of the cerebrospinal fluid (CSF) can be observed. Therefore, CSF analysis forms the basis for the ante mortem diagnostic evaluation of ruminants with clinical signs involving the CNS [2]. Especially in uncommon cases where a conclusive diagnosis cannot be established based on neurological examination alone, lumbar CSF analysis is a useful ancillary test for small ruminant practitioners [3]. Along with signalment, clinical signs, neurological examination, blood analysis, and imaging techniques, CSF analysis is crucial for the categorization and diagnosis of CNS diseases afflicting small ruminants with neurological signs. Since the CNS is not easily accessible in terms of clinico-pathological diagnostics, the evaluation of CSF is the most commonly used laboratory analysis for further characterizing CNS disease into inflammatory or non-inflammatory diseases [4]. Furthermore, it has been shown that CSF analysis is most sensitive in detecting inflammatory diseases in dogs [5]. Generally, changes in protein concentration or total leucocytes or differential cell counts can help to differentiate between inflammatory/infectious, neoplastic, parasitic, metabolic, and degenerative diseases in other species [6]. In addition, some disease conditions with primary lesions outside of the CNS can mimic neurologic disease, so CSF analysis can help clinicians exclude these diseases [6]. Moreover, serial CSF analysis might aid in monitoring treatment response. 

Knowledge of characteristic CSF findings in common CNS disorders is useful to clinicians in terms of managing the treatment of small ruminants with neurologic signs appropriately and promptly [4]. Despite overall good sensitivity in terms of detecting inflammatory changes in the CNS, CSF analysis is often not specific, and the analysis provides only further hints as to the presence or absence of a CNS disease. Further testing, namely, serology, culture, staining, and molecular techniques, such as polymerase chain reaction (PCR), can shed light on the causative agent. 

There is only a limited body of literature available relating to CSF analysis on ruminants. A retrospective study, which analyzed the CSF of 102 cows, showed that the analysis of CSF is an important tool for the diagnosis of bovine CNS diseases [4]. For small ruminants, only a few studies are available, with limited animal numbers [2,7,8,9]. Therefore, this study was initiated to retrospectively examine the associations between various CNS diseases and their corresponding CSF changes. CNS cases from sheep and goats published over the course of 14 years (2003–2016) were reexamined, and the results of CSF analysis were analyzed in relation to the definitive diagnosis (necropsy findings or clinical diagnosis) to define specific CSF patterns associated with different CNS diseases.

## 2. Materials and Methods

The selection of animals for the present study was performed in two steps. First, all CSF analysis results from goats and sheep between 2003 and 2016 were extracted from the databank of the Laboratory Information System (Vianova, Clinisys, Gent, Belgium) of the Clinical Laboratory, Vetsuisse Faculty of Zurich, Switzerland). In the second step, clinical records were collected and reviewed for all sheep and goats for which CSF analysis had been performed. The inclusion criteria were defined as follows: (1) sheep and goats with confirmed CNS disease and (2) sheep and goats with abnormal CSF findings without a specific CNS diagnosis. Animals without a specific CNS diagnosis were categorized as “undetermined”. A CNS disease diagnosis was either established based on clinical signs or necropsy findings. The following information was available for each animal: signalment (age, gender, and breed), general clinical examination results, specific neurological examination results, hematological and biochemical blood analysis results, and CSF analysis results. Age, sex, breed, castration status, and pre-treatment were not included in the exclusion criteria. The hematological and biochemical blood analyses included total red blood cell count, hematocrit, total white blood cell count, white blood cell differentiation, total protein, fibrinogen, bilirubin, urea, creatinine, liver values, creatine kinase, and electrolytes. The complete CSF analysis included protein concentration, red blood cell (RBC) count, total nucleated cell count (TNCC), and cytological evaluation of a cytospin specimen. 

CSF was collected either from the lumbosacral space (LS space) or the cisterna magna (CM). For the lumbosacral puncture, the animals were sedated with xylazine (Xylazin Streuli^®^ ad. us. vet., Uznach, Switzerland; dosage: 0.05–0.1 mg/kg, intramuscular) and then sternally positioned [10]. The puncture site was shorn, washed, disinfected, and locally anesthetized. The same procedure was applied for the collection from the CM, but the animal’s head was bent at a 90° angle to open up the intervertebral gap. To minimize the potential risk of spinal cord puncture, a study was performed on cows using ultrasound-assisted puncture; this study revealed that the animals’ heads could be bent less than 90° and that the structures could still be displayed optimally [11]. CSF was collected in a sterile plastic tube without anticoagulants (Milian SA, Geneva, Switzerland) or in EDTA tubes and immediately sent to the laboratory of the University Animal Hospital (Clinical Laboratory, Vetsuisse Faculty Zurich, Switzerland). All samples were analyzed within two hours of collection.

The basic diagnostic CSF analysis for each sample included macroscopic evaluation, TNCC and RBC counts, and the cytological evaluation of a cytospin specimen. Macroscopic evaluation of the CSF consisted of analyzing volume, transparency, and color. Furthermore, color was evaluated a second time after centrifugation. A Fuchs–Rosenthal hemocytometer was used to determine the TNCC after a 1:10 dilution of the native CSF with Samson’s Reagent (Kantonsapotheke Zürich, Zurich, Switzerland). RBC counting was performed with undiluted CSF in a Neubauer-improved hemocytometer. For cytological evaluation, a cytospin specimen was prepared (Shandon Cytospin 4, Thermo Fisher Scientific, Waltham, MA, USA), followed by modified Wright–Giemsa staining (Hema Tek 1000, Siemens, Munich, Germany). For cytological evaluation, a 100-cell differential was performed, if possible, and percentages were given for each leucocyte subpopulation. In cases where fewer than 100 cells were available, percentages were calculated based on the available cells from the cytospin specimen. Total protein was measured after centrifugation (five minutes at 390× *g* in a Hettich Rotina 32A centrifuge, Bäch, Switzerland) in the CSF supernatant using a colorimetric method (Pyrogallol method) from 2003 to July 2004. Starting in August 2004, a turbidimetric assay incorporating Benzethonium chloride as the precipitant was used (Total Protein Urine/CSF Gen.3, Roche Diagnostics, Rotkreuz, Switzerland) [12]. To differentiate between normal and abnormal CSF findings, cut-off values from the literature were applied. For sheep, TNCC < 6 cells/μL with only mononuclear cells and total protein concentration < 0.4 g/L were regarded to be within reference limits. For goats, TNCC < 5 cells/μL, with only mononuclear cells and total protein concentration < 0.7 g/L, were regarded as being within reference limits [10,13]. Based on the TNCC and the cytological evaluation of the CSF, the results were further divided into different types of pleocytosis (Table 1). Lymphocytic pleocytosis was defined as >70% lymphocytes; monocytic pleocytosis was defined as >70% monocytes; neutrophil pleocytosis was defined as >70% neutrophils; and eosinophil pleocytosis was defined as >20% eosinophils. Mixed mononuclear pleocytosis was defined as when the sum of lymphocytes and monocytes was >70%. If the proportions of lymphocytes, monocytes, and neutrophils were all individually less than 70%; those of lymphocytes and monocytes together were less than 70%; and the proportion of eosinophils was less than 20%, this was called mixed pleocytosis (Table 1) [14]. In addition, cytoalbuminologic dissociation was defined as TNCC within normal limits but with increased protein concentration, and neoplastic pleocytosis was registered if neoplastic cells or atypical lymphocytes were found.

## 3. Results

### 3.1. Characteristics of the Study Population

The age of the 44 sheep included in this study ranged from 2 months to 14 years; the age of the 27 goats ranged from 7 months to 14 years. There were forty-one female (93%) and three male (7%) sheep, and there were eight male (30%) and nineteen female (70%) goats. In the sheep, 26 of the 44 CSF samples (59%) were collected from the LS space and 7 (16%) were collected from the CM, and for 11 samples (25%), the collection site was not recorded. In goats, CSF was collected from the LS space in 16 of the 27 cases (59%), while eight samples (30%) were collected from the CM, and the collection site was not recorded for 3 goats (11%). In 54 of the 71 cases (76%), the final diagnosis was based on histopathological findings made during necropsy, while in 17 animals (24%), it was a clinical diagnosis since the treatment was successful and no histopathology was performed. Table 2 provides an overview of all CNS diseases that were observed in the study population. 

### 3.2. Sheep

Thirty-three of the forty-four sheep were diagnosed with infectious CNS diseases (75%), of which the majority were bacterial in origin (listeriosis 19/33; 58%). In four cases, there were non-infectious causes (9%), and in five cases, there were inflammatory causes (11%). In two cases, the cause of the disease was unknown (5%) (Table 2). 

The most common disease in sheep was listeriosis (19/44; 43%), followed by parasitic coenurosis (11/44; 25%) (Table 3). Listeriosis was diagnosed based on each patient’s medical history (fed silage) and clinical signs (unilateral facial paralysis or salivation) or based on the histopathologic necropsy findings. The median protein concentration in the CSF of sheep with confirmed listeriosis showed a marked increase compared to the reference interval (Table 3). However, there were also sheep with listeriosis and protein concentrations within the reference intervals. Eight sheep with listeriosis showed mixed mononuclear pleocytosis; four had a mixed form; four had a monocytic form; and three had lymphocytic pleocytosis (Table 3). 

The median protein concentration of the coenurosis cases in sheep was within normal limits, and four cases showed completely normal CSF. Besides that, two eosinophilic cases, three mixed mononuclear cases, one monocytic case, and one lymphocytic case of pleocytosis were found (Table 4). 

Of the two sheep with encephalitis, one had normal CSF, and one presented with moderate monocytic pleocytosis. In addition, one of the two meningoencephalitis cases in sheep presented moderate eosinophilic pleocytosis with only a mildly increased protein concentration. The second case showed mild cytoalbuminologic dissociation. 

Both sheep cases with nematodiasis had a mildly increased protein concentration and a moderately increased TNCC. One sheep presented lymphocytic pleocytosis, and a second one had eosinophilic pleocytosis. 

There was one sheep with lymphoma without CNS involvement. CSF analysis revealed high cytoalbuminologic dissociation. 

The sheep with bornavirus infection had a normal protein concentration but showed mild monocytic pleocytosis. The sheep case with encephalomalacia presented a markedly increased protein concentration and mild monocytic pleocytosis. 

The CSF from the sheep with necrosis of the hippocampus showed a normal TNCC but a mildly increased protein concentration, corresponding to mild cytoalbuminologic dissociation. Normal CSF was found in the sheep diagnosed with polioencephalomalacia. The sheep with myelitis also showed normal CSF after analysis. 

Two sheep showed altered CSF without a macroscopic or histological CNS disease being diagnosed in the pathology. For these animals, the category “undetermined” was used. One of these two sheep had mild cytoalbuminologic dissociation. The other showed a mildly increased protein concentration as well as a mildly increased TNCC. In the laboratory, this animal showed monocytic pleocytosis.

Among all sheep, the highest TNCC was observed in cases with listeriosis, followed by those with mengingoencephalitis. The lowest cell counts were observed in cases of myelitis, polioencephalomalacia, and necrosis hypocampus. Sheep with listeriosis also showed the highest protein concentration, followed by cases with lymphoma and encephalomalacia.

### 3.3. Goats

Fifteen of the twenty-seven goats were diagnosed with infectious CNS diseases (56%), of which the majority were bacterial in origin (listeriosis 9/15; 60%). For seven goats, there were non-infectious causes (26%), and in four cases, there were inflammatory causes (15%). In one case, the cause of the disease was unknown (4%) (Table 2). As with the sheep, the most common disease in goats was listeriosis (9/27; 33%). CSF analysis of goats with listeriosis revealed a median protein concentration of 0.94 g/L. All listeriosis cases showed abnormal CSF analysis results (Table 5). In three listeriosis cases, mixed mononuclear pleocytosis was observed. Two of the listeriosis cases showed a lymphocytic form, two showed a monocytic form, and two showed a mixed form of pleocytosis (Table 6). The second most common disease in goats in this study was nematodiasis (6/27; 22%). Three nematodiasis cases in goats showed lymphocytic pleocytosis, two revealed mixed mononuclear pleocytosis, and one presented eosinophilic pleocytosis. Four goats suffered from polioencephalomalacia. In all four cases, the CSF was shown to have a normal protein concentration, and in two cases a normal TNCC was observed as well. Consequently, 50% of the sheep with polioencephalomalacia had normal CSF; one goat had moderate lymphocytic pleocytosis and the other had mild mixed mononuclear pleocytosis. Of the three goats with meningoencephalitis, two were neutrophilic, and one had lymphocytic pleocytosis. The CSF from the goat with the cerebellum malacia showed a highly increased protein concentration and a moderately increased TNCC. Cytological analysis revealed monocytic pleocytosis. One goat with myelitis had moderate lymphocytic pleocytosis, wherein the protein concentration was normal. This goat also showed neoplastic pleocytosis due to a malignant lymphoma in an ileosacral lymph node, which infiltrated the lumbar spinal cord. 

One goat with degeneration of the white spinal cord matter had a normal protein concentration but showed a mildly increased TNCC. It was diagnosed with mild lymphocytic pleocytosis. 

The goat with subluxation between the sixth and seventh cervical vertebra showed a mildly increased protein concentration and a TNCC with mixed pleocytosis. 

There was one goat for which no diagnosis could be made, but lymphocytic pleocytosis was found in the laboratory. This goat was categorized as “undetermined”. In the laboratory, this goat showed a mildly increased protein concentration of 0.71 g/L, as well as a mildly increased TNCC.

Among all goats, the highest TNCC was observed in cases of meningoencephalitis and myelitis. Listeriosis in goats was paired with a lower TNCC compared to sheep.

## 4. Discussion

The central nervous system (CNS) is a complex tissue, and clinical signs alone are often not sufficient for a final diagnosis. Obtaining an accurate diagnosis among small ruminants is important because some neurologic diseases carry herd health implications or are zoonotic, so preventive measures are important for limiting or avoiding disease in at-risk populations [15]. CSF analysis is helpful in facilitating the diagnosis of specific diseases. Especially for the interpretation of inflammatory CNS diseases, CSF examination is very valuable and often, but not always, enables diagnosis [4]. 

This is the first study evaluating a larger number of CSF samples from small ruminants and correlating them with clinical or histopathological outcomes. In this study, premedication, age, sex, breed, or the castration status of the animals were not specifically considered. These variables could have a significant impact on the CSF results. 

Our results demonstrated that infectious diseases were the most common etiologies in both sheep and goats (48/71; 68%), and the most frequent cause of infection was bacteria (listeriosis 28/48; 58%). Serological tests or PCR analysis for viruses were rarely initiated and only if histopathology indicated suspicion of viral involvement. This may explain the low number of viral causes of CNS diseases in the studied population. 

The CSF results from sheep and goats with listeriosis were similar concerning the cytological pattern, and a predominance of mononuclear cells (monocytes or lymphocytes) was found. However, the types of pleocytosis varied. Lymphocytes (lymphocytic pleocytosis) or monocytes (monocytic pleocytosis) were the most prominent cell type, while in other cases, both cell types were found to be equally (mixed mononuclear pleocytosis) or neither cell-line-dominated (mixed pleocytosis). A similar study on cows showed that the majority of listeriosis cases appeared only as monocytic or mixed mononuclear pleocytosis [4]. Another comparable study on camelids with listeriosis also showed monocytic pleocytosis [16]. In contrast, two studies identified neutrophilic pleocytosis in small ruminants [17] and sheep [8] with listeriosis. One case report regarding a goat with neurolisteriosis showed marked neutrophilic pleocytosis with rare intracellular bacteria, which is consistent with Listeria monocytogenes [18]. The occurrence of various pleocytosis patterns in different studies and species with listeriosis can be attributed to different examination times at different stages of the disease and different degrees in terms of the severity of the disease or potential premedication. Unexpectedly, goats with listeriosis showed lower TNCCs than sheep. It can be speculated that sheep were presented later in the course of the disease due to herd husbandry, whereas goats were presented earlier with less severe signs of inflammation because they more often came from hobby farms. Both sheep and goats with listeriosis showed neurological signs like unilateral facial paralysis, salivation, and an inability to swallow. Sheep and goats generally have a poorer prognosis than cows [17]. Therefore, CSF analysis is the most useful antemortem diagnostic test, and the detection of pleocytosis in CSF from animals with these clinical signs supports a presumptive diagnosis of encephalitic listeriosis but does not provide a definitive diagnosis [17] since bacteriological culture is usually not performed due to low sensitivity [4].

The goat with subluxation between C6 and C7 showed mixed pleocytosis. Depending on the location and severity, trauma in the spinal cord can lead to different CSF changes and even produce normal CSF. Imaging methods such as X-rays, myelography, computed tomography, or magnetic resonance imaging are more suitable for diagnosis [19]. 

The CSF of the polioencephalomalacia cases in this study also presented different pleocytosis types. Four of the five polioencephalomalacia cases occurred in goats. Two of the affected goats, as well as one sheep with polioencephalomalacia, showed normal CSF results. The two remaining goats with polioencephalomalacia exhibited lymphocytic and mixed mononuclear pleocytosis. In calves with polioencephalomalacia, the CSF showed mononuclear pleocytosis or was unchanged, so it was concluded by the authors that the benefit of CSF analysis only lies in ruling out an infectious CNS disease, and CSF analysis does not appear to be the first choice in terms of a diagnostic tool for polioencephalomalacia [20]. In comparison, sheep with necrosis of the hippocampus showed cytoalbuminologic dissociation. The case with diagnosed encephalomalacia revealed a mild increase in TNCC and a monocytic pleocytosis. This can be compared to the mild mononuclear pleocytosis in the calves with polioencephalomalacia in the study conducted by Dore and Smith [20]. 

Coenurosis, a neurological parasitic infection, was only diagnosed in sheep and showed very variable CSF results. Four of eleven cases had normal CSF, two cases presented eosinophilic pleocytosis, and three cases showed mixed mononuclear pleocytosis. Five of the coenurosis cases presented with blood contamination, with RBC counts of >500/μL. Since iatrogenic blood contamination during CSF collection is known to influence the TNCC and corrective formulas are not recommended, the results of the five coenurosis cases with blood contamination must be interpreted with caution; instead, in pleocytosis, normal CSF should also be considered in these five cases. In the present study, the cut-off values for TNCC were only used to determine pleocytosis without considering the influence of possible blood contamination, and animals with marginally elevated TNCC and concomitant blood contamination were also classified in terms of pleocytosis type. Similar CSF patterns were found in a study evaluating cisternal CSF from sheep with chronic coenurosis [7]. 

Both sheep and goats with nematodiasis showed either lymphocytic or eosinophilic pleocytosis. In addition, there were also two mixed mononuclear pleocytosis cases among the goats. Therefore, the predominant cell types are mononuclear cells or eosinophils. Camelids, infected with parelaphostrongylus tenuis, also showed eosinophilic pleocytosis, with increased protein concentrations [16]. Like small ruminants, camelids suffer from hindquarters weakness, ataxia, and constipation. The sensorium is unadulterated, and an appetite usually exists [16]. Our findings concerning the presence of eosinophils or eosinophilic pleocytosis in small ruminant CSF are consistent with what has been described before in sheep with Coenurosis, where only 67% of all sheep with Coenurosis had eosinophilia in their CSF [7]. Therefore, eosinophilic pleocytosis can be absent in patients with a neurological parasitic infection. 

Interestingly, one sheep with multicentric lymphoma in the spleen, chest cavity, abdominal cavity, and different lymph nodes but without histopathology in the CNS should have had a normal TNNC and been free from neoplastic lymphocytes in the CSF but presented with a marked increase in total protein concentration. This is compatible with outflow dysfunction or intrathecal globulin production caused by a tumor. 

The goat with myelitis showed lymphocytic pleocytosis with atypical lymphocytes in the CSF analysis, and the sample was therefore well categorized as neoplastic pleocytosis. Upon necropsy, a malignant lymphoma in a lymph node of the ileosacral lymphatic center was found, which caused myelitis and neurological signs due to the infiltration of the lumbar spinal cord. This animal was constipated and showed reduced anal reflex as well as reduced panniculus reflex. For comparison, the sheep, which was also diagnosed with myelitis, presented with completely normal CSF (Table 3). 

There was a total of three cases in which no definitive diagnosis could be made (“undetermined”, Table 2) despite the fact that these animals had a pathological CSF. One goat had mild lymphocytic pleocytosis and a mildly increased protein concentration. One sheep had cytoalbuminologic dissociation, and the third case had mild monocytic pleocytosis. Since cytoalbuminologic dissociation was only triggered by a minimal increase in protein concentration, there was no certainty that there was a neurological problem because small increases in protein concentration can also be caused by disorders involving the blood–brain barrier or by blood contamination.

CSF reference values for cows vary from those of small ruminants. The cut-off values for TNCC (<10 TNCC/μL) are higher in cows, but protein concentrations in CSF are similar to those reported for small ruminants (0.67 g/L) [4]. 

Data from this study showed that mixed mononuclear pleocytosis was most common in sheep (12/44; 27%), followed by monocytic pleocytosis (9/44; 21%). In both situations, listeriosis was the most common underlying disease. Lymphocytic pleocytosis was the most frequently found abnormality in goats (10/27; 37%) with a variety of underlying diseases. The second most common type of pleocytosis in goats was mixed mononuclear pleocytosis (6/27, 22%), and half of these cases were caused by listeriosis. 

This study has several limitations due to its retrospective design, which are listed here: 1. In certain disease groups, sample numbers are very low; therefore, interpretation should be regarded with caution. 2. In cases where necropsy and histo-pathological examination were not performed and a final diagnosis was established based on clinical diagnosis, residual uncertainty remains. 3. Although this study population comprised two different sampling locations, all animals’ results were interpreted using cut-off values for TNCC and protein concentration for lumbar collection. 4. Iatrogenic blood contamination during sampling occurred in certain cases and disease groups, which might have affected pleocytosis type.

## 5. Conclusions

Based on the present study, it can be concluded that the most common cytologic patterns in small ruminants are monocytic and lymphocytic in origin with variable quantitative expression, whereas neutrophilic pleocytosis and cytoalbuminologic dissociation were rare findings. Infectious diseases of bacterial origin were the most common underlying causes of CSF alterations in sheep and goats, followed by parasitic disorders. The pleocytosis type is not helpful in terms of differentiating between disease types. Further testing (serology and PCR analysis) is therefore recommended to identify the causative infectious agents of CNS disorders.

## Figures and Tables

**Table 1:** Pleocytosis types used in the present study for interpretation of CSF.

Cell Type	Type of Pleocytosis					
	Lymphocytic	Monocytic	Neutrophilic	Eosinophilic	Mixed Mononuclear	Mixed
Lymphocytes	>70%				>70%	≤70%
Monocytes		>70%				≤70%
Neutrophils			>70%			≤70%
Eosinophils				>20%		≤20%

**Table 2:** Diseases in 71 small ruminants.

Disease	Cause of Disease	Diagnosis: Clinical (c)/ Pathological (p)	Sheep [n = 44]	Goats [n = 27]
Listeriosis	Infectious	10 (c)/18 (p)	19 (43%)	9 (33%)
Coenurosis	Infectious	10 (c)/1 (p)	11 (25%)	-
Nematodiasis	Infectious	1 (c)/7 (p)	2 (5%)	6 (22%)
Polioencephalomalacia	Non-infectious	4 (c)/1 (p)	1 (2%)	4 (15%)
Meningoencephalitis	Inflammatory	5 (p)	2 (5%)	3 (11%)
Encephalitis	Inflammatory	2 (p)	2 (5%)	-
Encephalomalacia	Non-infectious	1 (p)	1 (2%)	-
Degeneration of the white spinal cord matter	Non-infectious	1 (p)	-	1 (4%)
Subluxation C6-7	Non-infectious	1 (c)	-	1 (4%)
Lymphosarcoma	Non-infectious	1 (p)	1 (2%)	-
Borna	Infectious	1 (p)	1 (2%)	-
Myelitis	Inflammatory	2 (p)	1 (2%)	1 (4%)
Necrosis hippocampus	Non-infectious	1 (p)	1 (2%)	-
Cerebellum malacia	Non-infectious	1 (p)	-	1 (4%)
Undetermined	-	3 (p)	2 (5%)	1 (4%)

**Table 3:** CSF results from 44 sheep with CNS disease.

					Differential Cell Count (%)			
Disease Diagnosis	n (%)	Total Protein (RI 0.4 g/L)	RBC (RI 0/μL)	TNCC (RI < 6/μL)	Lymphocytes	Monocytes	Neutrophils	Eosinophils
Listeriosis	19 (43%)	1.71 (0.2–31.52)	1160.5 (31.25–10,920) n = 18	247 (10.33–9941.33)	31 (0–89)	45 (0–86)	11 (0–70)	0 (0–4)
Coenurosis	11 (25%)	0.27 (0.22–0.78)	1000 (0–40,000)	6.66 (0–49.3)	44 (22–90) n = 9	28 (8–75) n = 9	10 (0–18) n = 9	0 (0–44) n = 9
Encephalitis	2 (5%)	0.575 (0.21–0.94)	50 (30–70)	33.165 (4–62.33)	18.565 (13.13–24)	77.4 (74–80.8)	3.525 (2–5.05)	0.505 (0–1.01)
Meningoencephalitis	2 (5%)	0.445 (0.43–0.46)	1585 (0–3170)	233.9 (3.8–464)	67 n = 1	0 n = 1	0 n = 1	33 n = 1
Nematodiasis	2 (5%)	0.64 (0.64–0.64)	4053.5 (367–7740)	75.95 (68.6–83.3)	79 (68–90)	1 (1–1)	0 (0–0)	20 (9–31)
Lymphoma	1 (2%)	3.16	660	4.66	43	54	3	0
Borna	1 (2%)	0.37	10	6.33	18	82	0	0
Encephalomalacia	1 (2%)	2.58	1210	7.33	9	90	1	0
Necrosis hippocampus	1 (2%)	0.6	112.5	1.83	No data	No data	No data	No data
Polioencephalomalacia	1 (2%)	0.14	320	0.67	No data	No data	No data	No data
Myelitis	1 (2%)	0.17	45	0.83	No data	No data	No data	No data
Undetermined	2 (5%)	0.445 (0.44–0.45)	1747.5 (565–2930)	9.665 (2.33–17)	12 n = 1	74 n = 1	14 n = 1	0 n = 1

**Table 4:** CSF results from 44 sheep with CNS diseases.

				Pleocytosis						
Disease Diagnosis	n (%)	Normal	Cytoalbuminologic Dissociation	Lymphocytic	Monocytic	Neutrophilic	Eosinophilic	Mixed Mononuclear	Mixed	Neoplastic
Listeriosis	19 (43%)	0 (0%)	0 (0%)	3 (16%)	4 (21%)	0 (0%)	0 (0%)	8 (42%)	4 (21%)	0 (0%)
Coenurosis	11 (25%)	4 (36%)	0 (0%)	1 (9%)	1 (9%)	0 (0%)	2 (18%)	3 (27%)	0 (0%)	0 (0%)
Encephalitis	2 (5%)	1 (50%)	0 (0%)	0 (0%)	1 (50%)	0 (0%)	0 (0%)	0 (0%)	0 (0%)	0 (0%)
Meningoencephalitis	2 (5%)	0 (0%)	1 (50%)	0 (0%)	0 (0%)	0 (0%)	1 (50%)	0 (0%)	0 (0%)	0 (0%)
Nematodiasis	2 (5%)	0 (0%)	0 (0%)	1 (50%)	0 (0%)	0 (0%)	1 (50%)	0 (0%)	0 (0%)	0 (0%)
Lymphoma	1 (2%)	0 (0%)	1 (100%)	0 (0%)	0 (0%)	0 (0%)	0 (0%)	0 (0%)	0 (0%)	0 (0%)
Borna	1 (2%)	0 (0%)	0 (0%)	0 (0%)	1 (100%)	0 (0%)	0 (0%)	0 (0%)	0 (0%)	0 (0%)
Encephalomalacia	1 (2%)	0 (0%)	0 (0%)	0 (0%)	1 (100%)	0 (0%)	0 (0%)	0 (0%)	0 (0%)	0 (0%)
Necrosis hippocampus	1 (2%)	0 (0%)	1 (100%)	0 (0%)	0 (0%)	0 (0%)	0 (0%)	0 (0%)	0 (0%)	0 (0%)
Polioencephalomalacia	1 (2%)	1 (100%)	0 (0%)	0 (0%)	0 (0%)	0 (0%)	0 (0%)	0 (0%)	0 (0%)	0 (0%)
Myelitis	1 (2%)	1 (100%)	0 (0%)	0 (0%)	0 (0%)	0 (0%)	0 (0%)	0 (0%)	0 (0%)	0 (0%)
Undetermined	2 (5%)	0 (0%)	1 (50%)	0 (0%)	1 (50%)	0 (0%)	0 (0%)	0 (0%)	0 (0%)	0 (0%)

**Table 5:** CSF results from 27 goats with CNS disease.

					Differential Cell Count (%)			
Disease Diagnosis	n (%)	Total Protein (RI < 0.7 g/L)	RBC (RI 0/μL)	TNCC (RI < 5/μL)	Lymphocytes	Monocytes	Neutrophils	Eosinophils
Listeriosis	9 (33%)	0.94 (0.26–2.81)	1450 (81.3–35,400)	54.33 (9–925.33)	53 (0–78)	27 (0–80)	11 (0–48)	0 (0–8)
Nematodiasis	6 (22%)	0.84 (0.38–24)	48.375 (5–2515)	55.915 (12.91–352)	70.5 (23–91)	11.5 (1–29)	1.5 (0–9)	10.5 (0–75)
Cerebrospinal necrosis	4 (15%)	0.255 (0.21–0.38)	732.5 (62.5–2100)	5.93 (0.33–100)	71 (50–100) N = 3	0 (0–50) N = 3	0 (0–29) N = 3	0 (0–0) N = 3
Meningoencephalitis	3 (11%)	3.33 (0.57–13.16)	1835 (192.5–6020)	2790 (20.33–13,170)	9 (5–86)	2 (0–13)	89 (1–95)	0 (0–0)
Cerebellum malacia	1 (4%)	15.14	30	145	0	93	7	0
Myelitis	1 (4%)	0.58	1190	244	98	1	1	0
Degeneration of the white spinal cord matter	1 (4%)	0.25	465	7	98	1	1	0
Subluxation C6-7	1 (4%)	0.71	13,640	8.83	31	35	28	6
Undetermined	1 (4%)	0.71	925	11	15	15	6	0

**Table 6:** Classification of CSF from 27 goats with CNS disease.

				Pleocytosis						
Disease Diagnosis	n (%)	Normal	Cytoalbuminologic Dissociation	Lymphocytic	Monocytic	Neutrophilic	Eosinophilic	Mixed Mononuclear	Mixed	Neoplastic
Listeriosis	9 (33%)	0 (0%)	0 (0%)	2 (22%)	2 (22%)	0 (0%)	0 (0%)	3 (33%)	2 (22%)	0 (0%)
Nematodiasis	6 (22%)	0 (0%)	0 (0%)	3 (50%)	0 (0%)	0 (0%)	1 (17%)	2 (33%)	0 (0%)	0 (0%)
Polioencephalomalacia	4 (15%)	2 (50%)	0 (0%)	1 (25%)	0 (0%)	0 (0%)	0 (0%)	1 (25%)’	0 (0%)	0 (0%)
Meningoencephalitis	3 (11%)	0 (0%)	0 (0%)	1 (33%)	0 (0%)	2 (67%)	0 (0%)	0 (0%)	0 (0%)	0 (0%)
Cerebellum malacia	1 (4%)	0 (0%)	0 (0%)	0 (0%)	1 (100%)	0 (0%)	0 (0%)	0 (0%)	0 (0%)	0 (0%)
Myelitis	1 (4%)	0 (0%)	0 (0%)	1 (100%)	0 (0%)	0 (0%)	0 (0%)	0 (0%)	0 (0%)	1 (100%)
Degeneration of the white spinal cord matter	1 (4%)	0 (0%)	0 (0%)	1 (100%)	0 (0%)	0 (0%)	0 (0%)	0 (0%)	0 (0%)	0 (0%)
Subluxation C6-7	1 (4%)	0 (0%)	0 (0%)	0 (0%)	0 (0%)	0 (0%)	0 (0%)	0 (0%)	1 (100%)	0 (0%)
Undetermined	1 (4%)	0 (0%)	0 (0%)	1 (100%)	0 (0%)	0 (0%)	0 (0%)	0 (0%)	0 (0%)	0 (0%)

## Data Availability

Data are contained within the article and Supplementary Materials.

## Supplementary Materials

The following supporting information can be downloaded at: https://www.mdpi.com/article/10.3390/ani14010046/s1, Table S1: raw data sheep; Table S2: raw data goats.
